# Temporal and spatial detection of *Candidatus* Liberibacter asiaticus putative effector transcripts during interaction with Huanglongbing-susceptible, −tolerant, and -resistant citrus hosts

**DOI:** 10.1186/s12870-019-1703-4

**Published:** 2019-04-02

**Authors:** Qingchun Shi, Marco Pitino, Shujian Zhang, Joseph Krystel, Liliana M. Cano, Robert G. Shatters, David G. Hall, Ed Stover

**Affiliations:** 1 U.S. Horticultural Research Laboratory, Agricultural Research Service, United States Department of Agriculture, Fort Pierce, FL USA; 2 Institute of Food and Agricultural Sciences, Department of Plant Pathology, Indian River Research and Education Center, University of Florida, Fort Pierce, FL USA

## Abstract

**Background:**

Citrus Huanglongbing (HLB) is a bacterial disease with high economic significance. The associated agent *Candidatus* Liberibacter asiaticus is a fastidious, phloem-limited, intracellular bacterium that is transmitted by an insect vector the Asian citrus psyllid (ACP). The genome of *Ca.* L. asiaticus contains protein secretion machinery that suggests host cell modulation capacity of this bacterium.

**Results:**

A total of 28 candidate effectors, an important class of secreted proteins, were predicted from the Ca. *L. asiaticus* genome. Sequence specific primers were designed for reverse transcription (RT) and quantitative PCR (qPCR), and expression was validated for 20 of the effector candidates in infected citrus with multiple genetic background. Using detached leaf inoculation, the mRNA of effectors was detected from 6 h to 7 days post ACP exposure. It was observed that higher bacterial titers were associated with a larger number of effectors showing amplification across all samples. The effectors’ expression were compared in citrus hosts with various levels of HLB tolerance, including susceptible Duncan grapefruit and Washington navel orange, tolerant citron and Cleopatra mandarin, and resistant Pomeroy trifoliate and Carrizo citrange. Across all genotypes relatively high expression was observed for *CLIBASIA_03695*, *CLIBASIA_00460*, *CLIBASIA_00420*, *CLIBASIA_04580*, *CLIBASIA_05320*, *CLIBASIA_04425*, *CLIBASIA_00525* and *CLIBASIA_05315* in either a host-specific or -nonspecific manners. The two genotypes in each HLB-response group also show effector-expression profiles that seem to be different. In a companion study, the expression of effectors was compared between leaves and roots of own-rooted citrus that had been Ca. *L. asiaticus*-infected for more than a year. Results indicated relatively high expression of *CLIBASIA_03875*, *CLIBASIA_04800* and *CLIBASIA_05640* in all leaf and some root tissues of citron, Duncan and Cleopatra.

**Conclusion:**

This temporal and spatial expression analysis of Ca. *L. asiaticus* effectors identified candidates possibly critical for early bacterial colonization, host tolerance suppression and long-term survival which are all worthy of further investigation.

**Electronic supplementary material:**

The online version of this article (10.1186/s12870-019-1703-4) contains supplementary material, which is available to authorized users.

## Background

Huanglongbing (HLB) is currently the most devastating disease among citrus crops, significantly impacting the production in the United States and the industry worldwide. In the U.S. it is associated with the bacterium *Candidatus* Liberibacter asiaticus (Ca. *L. asiaticus*). Ca. *L. asiaticus* is an α-proteobacterium restricted in citrus to the phloem cells and is transmitted by the insect vector Asian citrus psyllid (ACP, *Diaphorina citri*). Likely associated with its intracellular nature, Ca. *L. asiaticus* is fastidious and it has not been maintained in sustainable culture in axenic conditions [1, 2]. Therefore, Koch’s Postulate remains incomplete for this pathogen and studies related to bacterial pathogenesis are especially challenging. However, complete genome sequences of several Ca. *L. asiaticus* isolates have been reported and made publicly available [3–6]. Sequence data has provided useful genetic information on the evolution of the bacterium and permitted inferences on virulence mechanisms and essential metabolism. The genomic dataset includes many putative genes that can be subject to reverse genetics for functional analysis.

Plants have evolved layered innate immune systems that function via inter- and intracellular defenses [7]. Cell surface receptors can perceive pathogen-derived molecules such as pathogen-associated molecular patterns (PAMPs) which then activate PAMP-triggered immunity (PTI). In turn, pathogens secrete effector proteins that suppress PTI or regulate plant physiology to facilitate disease development, which results in effector-triggered susceptibility (ETS). Incompatible hosts maintain resistance (R) genes capable of recognizing the effectors and then causing rapid immune responses categorized as effector-triggered immunity (ETI). In the Ca. *L. asiaticus* genome, a set of flagellum-associated genes have been identified [3] including the *Fla* gene which includes the 22-amino acid PAMP flg22 [8]. Assays with citrus hosts indicated Ca. *L. asiaticus* flg22 elicited plant defenses that differed in strength between susceptible and tolerant citrus suggesting this may have a role in disease tolerance [9]. In the study of Ca. *L. asiaticus* effectors, *in silico* genome searches uncovered a repertoire of candidates [10, 11]. Protein function studies revealed that the effector CLIBASIA_05315 induced ETI-like reactions in *Nicotiana benthamiana* [11] and contributed to excessive cellular starch accumulation, a typical physiological disorder associated with HLB [12]. Recently, a papain-like cysteine protease was identified to be the target of CLIBASIA_05315 in citrus and this uncovered an interesting aspect of virulence mechanism for HLB [13].

Effector biology is emerging as an important aspect of the investigation on plant-pathogen interactions, as secreted effector proteins play many roles in the pathogenicity that lead to initial infection and host colonization. An understanding of fundamental effector biology is key to revealing pathogen evolution to achieve virulence, including subcellular localizations, mechanisms of host cell modulation, and *in planta* binding targets including susceptible and R genes [14–16]. Importantly, characterization of effectors provides guidance on and accelerates crop resistance breeding. In combating potato late blight caused by *Phytophthora infestans*, increasing numbers of R gene-effector pairs have been identified [17, 18] and implemented in breeding to select for naturally-occurring R genes that provide durable resistance [19]. Alternatively, R genes identified from biochemical or genetic approaches can be cloned and used in transgenic production of resistant cultivars, a strategy that has been demonstrated in tomato [20], rice [21], potato [22] and alfalfa [23] as examples.

Genetic resistance to HLB appears to be lacking within the *Citrus* genus. However, disease tolerance as shown by mild symptom and less impairment on tree development have been observed in both controlled inoculations [24, 25] and field pathogen exposure [26–28]. In Florida, an evaluation of 83 *Citrus* and *Citrus* relatives accessions with 6-years of field exposure of HLB identified citron (*Citrus medica*) as a genetic source of tolerance [28]. The *Citrus* relatives trifoliate orange (*Poncirus trifoliata*) and its hybrids have been an important resource as rootstocks and showed marked HLB tolerance/resistance in multiple studies [27, 29, 30]. On the other hand, commercially important citrus types such as grapefruits (*C. paradisi*), sweet oranges (*C. sinensis*) and mandarins (*C. reticulata*) are all negatively impacted by HLB, but disease severity varies greatly. Several studies report potentially useful tolerance in some mandarins [24, 26, 31], which also appears to be heritable (Stover, unpublished results).

In this study, we conducted *in silico* analysis of the Ca. *L. asiaticus* genome and identified a total of 28 effector candidates. The mRNA of 20 effectors were detected in Ca. *L. asiaticus*-infected citrus by RT-qPCR. Using a detached leaf assay for insect-mediated bacterial transmission, effector mRNA could be detected from 6 h to 7 days after ACP infestation, and the number of effectors detected was positively correlated with the bacterial titer. Subsequently, the expression of effectors was compared in six citrus types with different HLB tolerance levels, including citron, Duncan grapefruit, Cleopatra mandarin, Pomeroy trifoliate, Washington navel orange and Carrizo citrange. Results indicated some effector candidates had relatively high expression in multiple citrus genotypes regardless of tolerance levels, while other effectors had host-specific expression patterns. The effectors expression was also compared between leaf and root tissues in own-rooted citrus which had been infected with Ca. *L. asiaticus* for more than a year. Several candidate effectors were found to have relatively high transcriptional level in leaf tissue and some had higher expression in roots. Taken together, these studies provide guiding information on Ca. *L. asiaticus* utilization/deployment of effectors at early- and late-colonization stages and different tissues and hosts, which may lead to the selection of promising candidates for functional analysis and direct citrus resistance breeding.

## Results

### Ca. *L. asiaticus* Secretome analysis for effector candidate identification

**Fig. 1:**
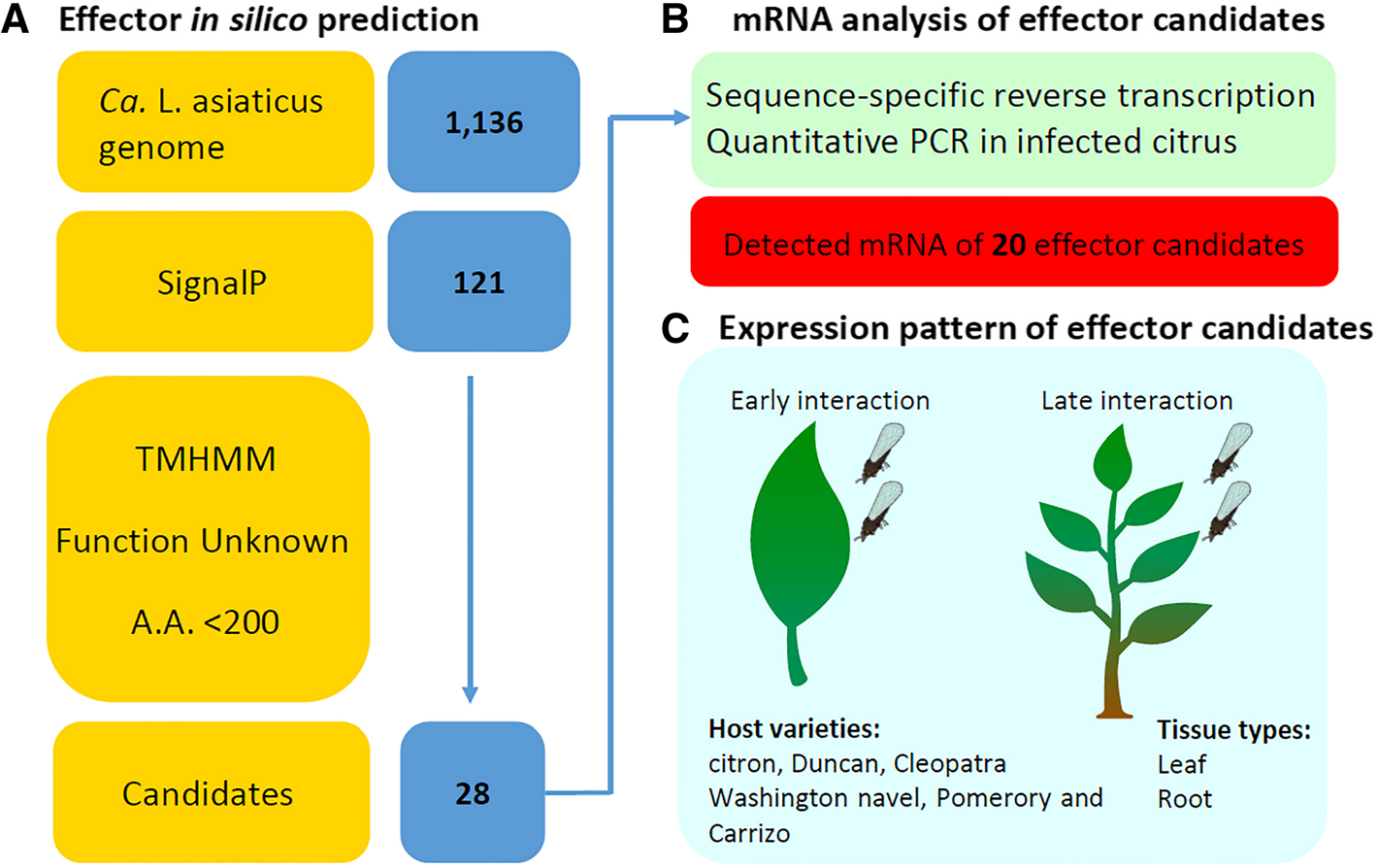
The flow chart of the study on expression of Ca. *L. asiaticus* effectors in citrus hosts. (**a**) The bioinformatics pipeline was applied to identify secreted candidate effectors using 1136 CDS from Ca. *L. asiaticus* genome. (**b**) Preliminary detection of mRNA using RT-qPCR measured expression of 20 candidates in infected citrus. (**c**) Effector expression pattern analysis during early and late bacterial-host interactions, in different citrus genotypes and between different tissue types

The Ca. *L. asiaticus* psy62 v1 genome (Genbank accession NC_012985.3) [3] was used for bioinformatic prediction of effectors. Based on common features of bacterial secreted proteins, the 1136 protein-coding sequences were filtered using SignalP [32] to predict the presence of signal peptides (SP) (Additional file 1: Table S1A) which were then subjected to TMHMM v2 [33, 34] to eliminate proteins with transmembrane domains [35]. Subsequently, the candidates with fewer than 200 amino acids were selected, and homology was searched using the National Center for Biotechnology Information (NCBI) BLAST tool. The proteins without any annotated homologs were analyzed in this study. A group of 28 putative Ca. *L. asiaticus* effectors was identified and used for the following experiments (Additional file 1: Table S1B and Fig. 1).

### Detection of putative effectors by RT-qPCR in the multiple citrus genotypes infected by Ca. *L. asiaticus*

Sequence specific primers (SSPs) were designed for both reverse transcription and qPCR for greater specificity and higher capacity in amplification of effectors at low enrichment. Primers for reverse transcription were chosen at the 3′ end of the mRNAs to direct the synthesis of the first strand of cDNA. Forward and reverse primers were designed downstream of the cDNA synthesis primer binding region (Additional file 2: Figure S1). As one of the objectives was to determine if the expression pattern of effectors differed between HLB -susceptible, −tolerant and -resistant citrus, the SSPs efficacy were tested using multiple citrus species where specificity was defined as positive amplification in the Ca. *L. asiaticus*-infected tissue but not when uninfected. Hence RNA was isolated from Ca. *L. asiaticus* positive and negative leaves of citron, Duncan grapefruit and Cleopatra mandarin, Washington navel, Pomeroy trifoliate and Carrizo citrange for cDNA synthesis and qPCR. The SSPs of 20 effector candidates showed Ca. *L. asiaticus* specific amplification of targets in all citrus genotypes (Fig. 1 and Additional file 3: Table S2), whereas the remaining amplified in at least one of the uninfected citrus genotypes.

### Effector expression during early Ca. *L. asiaticus*-citrus interactions and bacterial titer dependent effector mRNA detection

**Fig. 2:**
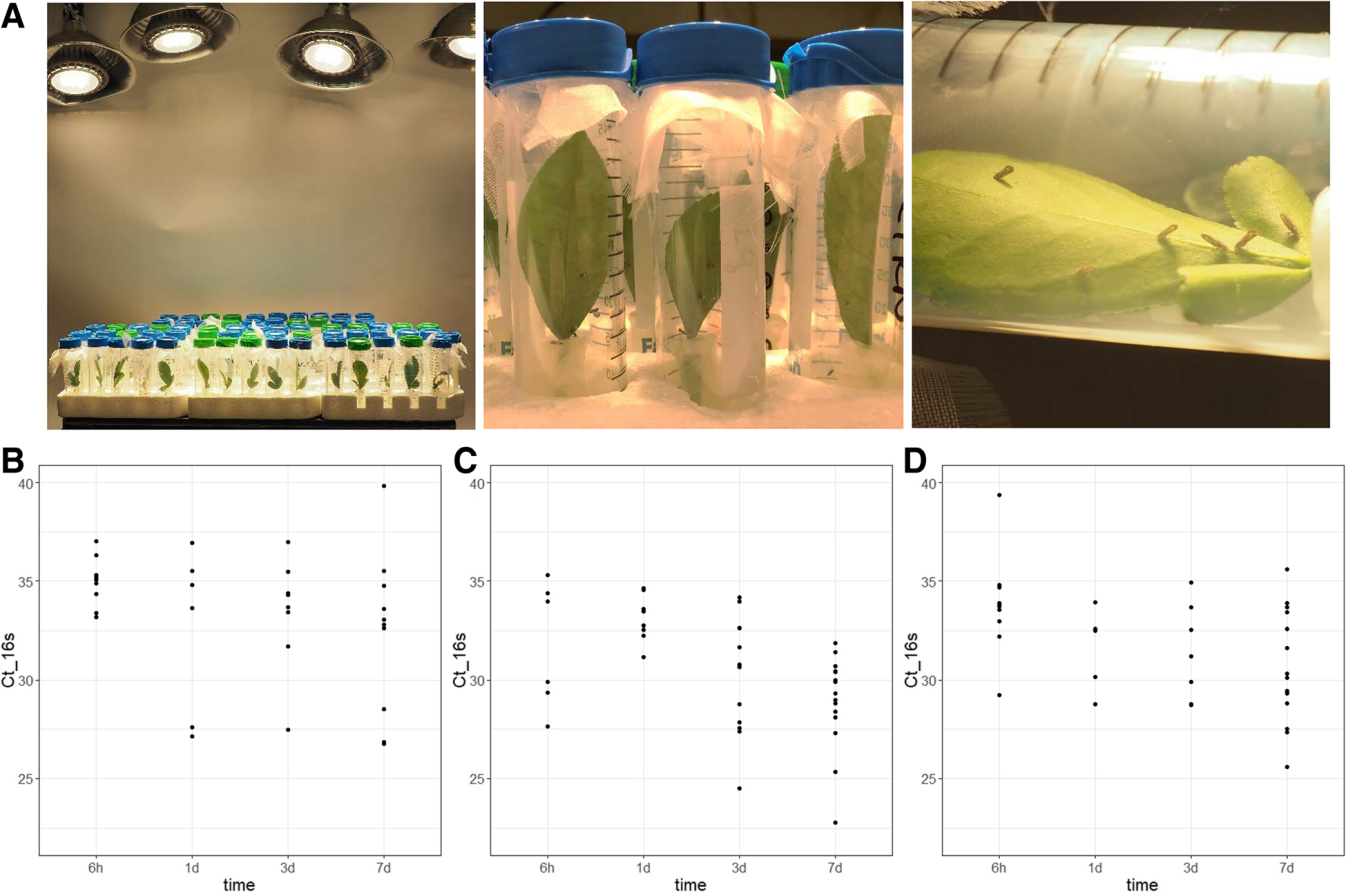
Photographs of detached leaf inoculation using Ca. *L. asiaticus*-infected ACP (**a**) and the distribution of bacterial titer measured from leaf samples collected at 6 h, 1d, 3d and 7d post ACP exposure (**b**-**d**). The experiments were conducted using leaves from citron (**b**), Duncan (**c**) and Cleopatra (**d**). The bacterium was quantified by qPCR amplifying the *16 s* rDNA with Las-long primer from 100 ng of citrus DNA template. The cycle threshold values (C_t_) were used to generate the dot plots for each time point

Due to the fastidious nature of this organism, common bacterial inoculation methods cannot be used to study the early interactions between Ca. *L. asiaticus* and citrus. This study used ACP infestation with detached leaves as previously described for inoculation [36] (Fig. 2a) to study an early infection time course. To compare Ca. *L. asiaticus* effector expression in citrus genotypes with different HLB tolerance levels, citron, Duncan, and Cleopatra citrus were used. Leaves from each genotype were exposed to ACP feeding for 6 h (h), 1, 3 and 7 days (d). Midrib DNA analysis indicated some successful bacterial transmissions at all of the time points and citrus genotypes, which provided multiple Ca. *L. asiaticus* positive samples for effector expression study (Fig. 2b). Three randomly selected infected samples at each time point and genotype were used for RT-qPCR, and showed detection of the mRNA of effectors at all the time points and genotypes studied (Additional file 4: Table S3). The number of effectors detected by RT-qPCR was generally low in most of samples which made it difficult to apply mean calculations and statistics; although some effector showed expression across more than two time points such as ‘4025’ in citron and ‘5315’ in Duncan and Cleopatra (From here on the last 4 digits of Ca. *L. asiaticus* gene locus name are used for simplicity). Noticeably, two samples with higher bacterial titers at 6 h and 3d in citron provided detectable mRNA of larger number of effectors (Additional file 4: Table S3), which led to a hypothesis that bacterial titer and number of effectors detected by RT-qPCR are positively correlated.

**Fig. 3:**
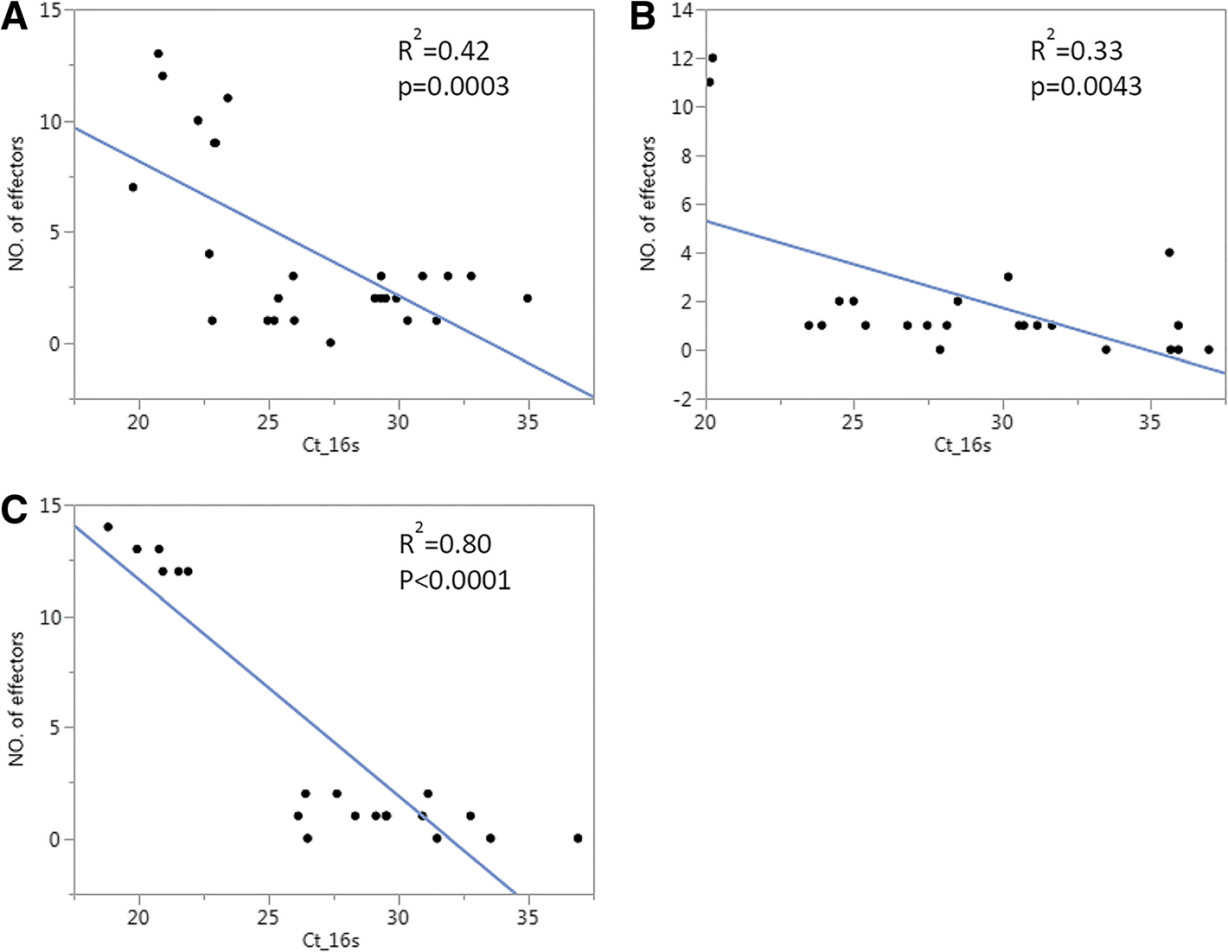
A linear relationship between the numbers of effectors detected by RT-qPCR and Ca. *L. asiaticus* titer in citron (**a**), Duncan (**b**) and Cleopatra (**c**). The calculation used samples from the detached leaf assay collected at 6 h, 1, 3 and 7 d post ACP exposure, and from leaf samples collected at 2, 4, 6, 8 and 10 wk after ACP inoculation to the citrus plants (Shi et al., 2017). The data analysis was done by JMP Genomics Fit Y by X function

To test this hypothesis, additional Ca. *L. asiaticus*-infected samples at a later infection stage were included for a larger dataset and more diverse sample pools. These RNA samples were generated from a previous study on citrus transcriptome response to Ca. *L. asiaticus* at 2, 4, 6, 8 and 10 weeks (wk) after inoculation [9], from which multiple samples from each time point were used for effector mRNA detections. From the RT-qPCR data, the number of effectors expressed was counted and plotted against the C_t_ values quantifying Ca. *L. asiaticus 16 s* in each sample and each genotype. The results indicated a significant linear relationship between the two groups of variables in the three citrus genotypes tested, where the liner model fit was highest in Cleopatra (R^2^ = 0.80) and lowest in Duncan (R^2^ = 0.33) (Fig. 3a-c).

### Differential effector expression in citrus species with various HLB tolerance

**Fig. 4:**
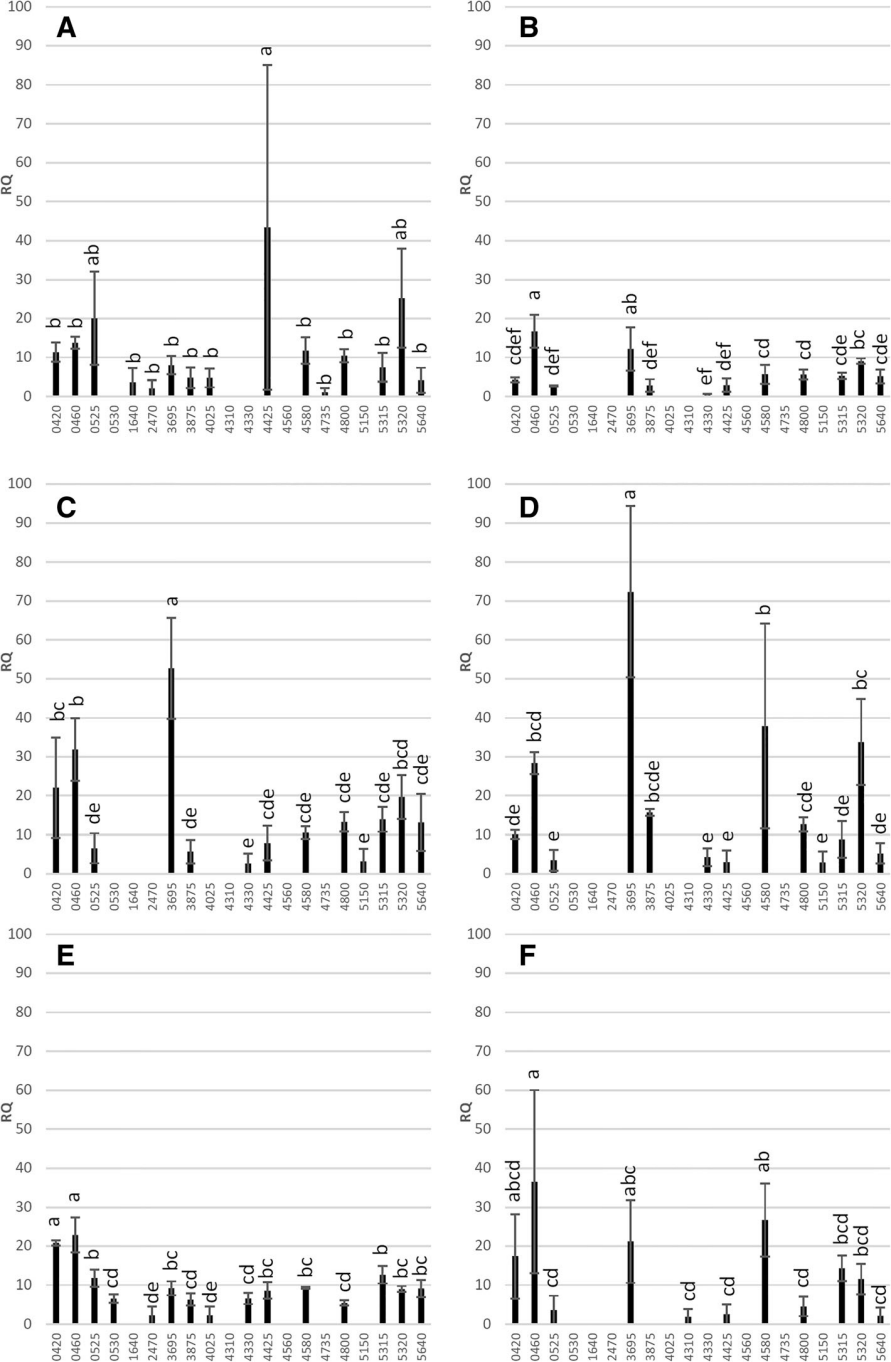
The expression of Ca. *L. asiaticus* effectors in Duncan (**a**), Washington navel (**b**), citron (**c**), Cleopatra (**d**), Pomeroy (**e**), and Carrizo (**f**) after 7-day ACP infestation in a detached leaf assay. Three randomly selected Ca. *L. asiaticus* positive leaves were used for RNA isolation and expression analysis. The C_t_ values of each effector generated by RT-qPCR were normalized with citrus endogenous control *UPL7* and transformed into relative quantification (RQ) values (2^-ΔCt^). The effectors with expression detections were subject to pair-wise comparison of standard least-square means (LS means) with Student’s t-test (*p* < 0.05). Expression levels indicated with the same letter are not significantly different. Bars are means ± standard error (*n* = 3)

To determine if effector expression patterns are different between HLB tolerant, susceptible, and resistant hosts at the same infection stage, the detached leaf study used in two highly susceptible citrus genotypes (Duncan and Washington navel orange), two tolerant genotypes (Cleopatra and citron), and two resistant genotypes (Pomeroy trifoliate and Carrizo citrange). Leaves of the six citrus genotypes were exposed to a 7-day infestation by the same ACP population for bacterial inoculation and only leaves which were Ca. *L. asiaticus* positive were used for effector analysis. The relative quantification (RQ) of effectors was compared within each citrus type to identify the higher expressed candidates that were likely to be critical for virulence. In the susceptible Duncan, only ‘4425’ had high expression while the level of other detected effectors were similar (Fig. 4a). Effectors ‘0460’ and ‘3695’ were the most expressed effectors in Washington Navel (Fig. 4b). In citron ‘3695’ showed the highest expression among all the effectors, and ‘0460’ and ‘0420’ were also highly ranked (Fig. 4c). Similarly ‘3695’ was the highest expressed effector in Cleopatra, and ‘4580’ and ‘5320’ had higher expression than most of the others (Fig. 4d). The trifoliate orange Pomeroy showed ‘0420’ and ‘0460’ as the highest expressing group, followed by ‘0525’ and ‘5315’ as the next group (Fig. 4e). In Carrizo, ‘0460’ and ‘4580’ showed significant higher levels than some other effectors, while the rest had no difference from each other (Fig. 4f). Therefore, we noted genotype-specific high expression of ‘4425’ in the susceptible Duncan, and genotype non-specific expression of ‘3695’ and ‘0460’ in multiple citrus. The two genotypes in each HLB-response group also show effector-expression profiles that seem to be different from each other.

### Differential effector expression between leaf and root tissues in Ca. *L. asiaticus*-infected citrus

**Fig. 5:**
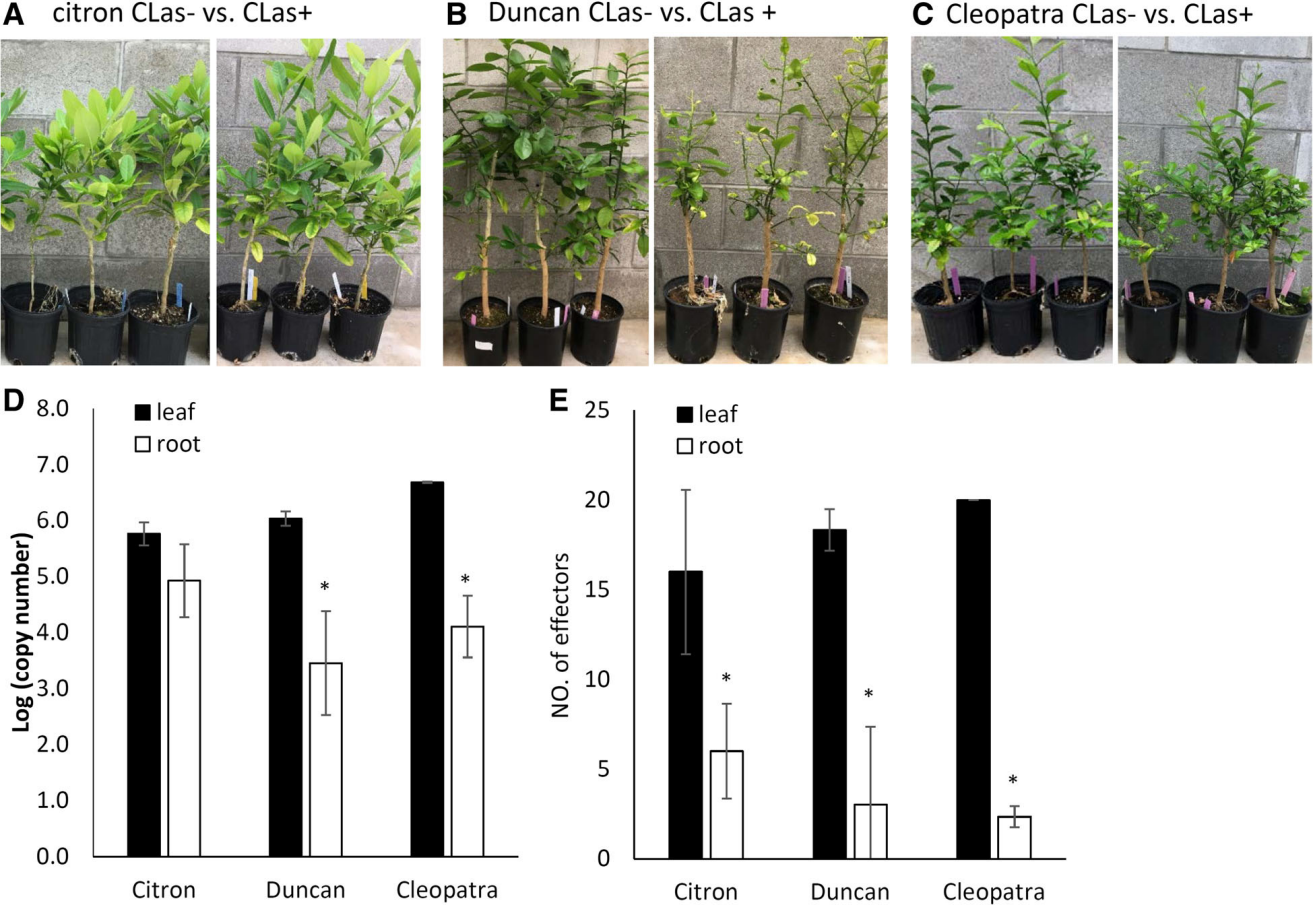
Comparison of effector expression in the leaf and root of citron (**a**), Duncan (**b**) and Cleopatra (**c**) infected by Ca. *L. asiaticus*. Photographs display the health of Ca. *L. asiaticus*-infected plants (**a**-**c**) over a year after inoculation (right), in comparison to their uninfected controls (left). The Ca. *L. asiaticus* was quantified by qPCR amplifying *16 s* rDNA from 100 ng of DNA template. A standard curve method with the function Log (copy number) = − 0.289*C_t_ + 11.66 was used to calculate *16 s* copy numbers (**d**). The RNA was isolated from the same group of samples for effector analysis by RT-qPCR, from which the number of effectors detected was compared between leaf and root tissues in citron, Duncan and Cleopatra (E). Analysis was based on three biological replicates and was analyzed by Student’s t-test (*p* < 0.05) and significant difference between leaf/root pairs is indicated by an asterisk

It is well known that infection of Ca. *L. asiaticus* occurs in citrus roots, which causes damage that directly affects tree health and also serves as source of inoculum to new foliar flushes through vascular movement of the bacterium [37]. Comparative analysis of the effector expression in leaf and root was conducted to study if effector expression has a tissue-specific pattern suggesting the bacterium employs different pathogenic strategies to colonize above- and below-ground plant organs. Ca. *L. asiaticus*-infected own-rooted citron, Duncan, and Cleopatra were used that had inoculated by ACP infestation in 2016 [9]. All plants were tested Ca. *L. asiaticus* positive in 2017 and after one year tolerant citron and Cleopatra plants were symptomatic but still maintained similar growth to their uninfected controls (Fig. 5a and c), whereas Duncan plants showed stunted growth and branch dieback (Fig. 5b).

**Fig. 6:**
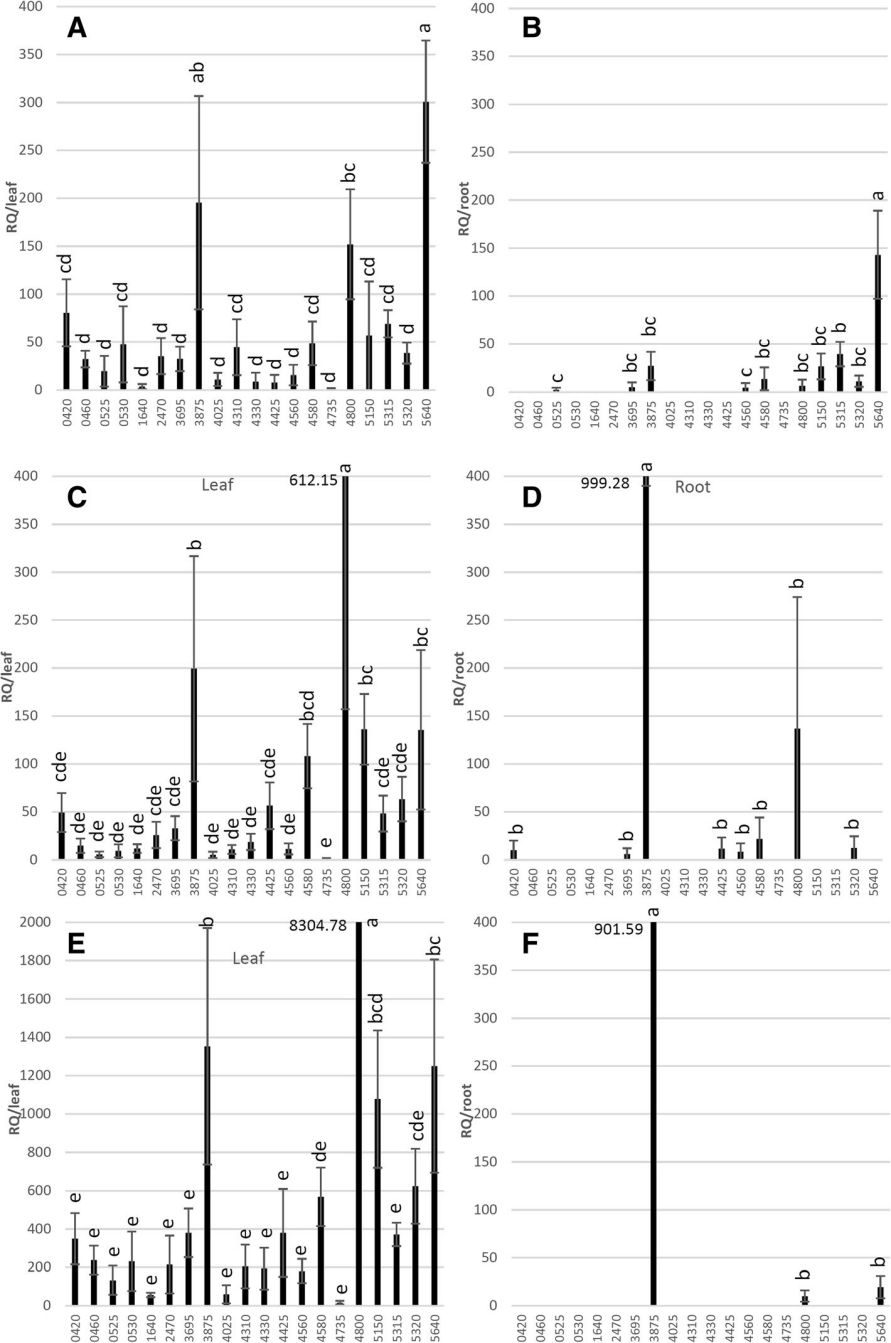
Comparison of effector expression in the leaf and root tissues of citron (**a** and **b**), Duncan (**c** and **d**) and Cleopatra (**e** and **f**). Leaf and root samples were collected from three plants of each citrus genotype that were inoculated 14–17 months prior via ACP infestation. In the expression analysis, the C_t_ values of each effector generated by RT-qPCR were normalized with citrus endogenous control *UPL7* and transformed into relative quantification (RQ) values (2^-delatCt^). The effectors with expression detected were subject to pair-wise comparison of standard least-square means (LS means) with Student’s t-test (*p* < 0.05). Expression levels indicated with the same letter are not significantly different. Bars are means ± standard error (*n* = 3)

Bacterial titers determined by *16 s* quantification were lower in the roots of Duncan and Cleopatra than leaves, although this difference was insignificant in citron (Fig. 5d). Correspondingly, RT-qPCR analysis of effectors indicated there were lower numbers of effectors detected in roots than in leaves in all of the citrus types (Fig. 5e), which is in agreement with the previous observation on titer dependent effector mRNA detection (Fig. 3). For the effectors with detected mRNA, in citron ‘5640’ was the highest expressed effectors in both leaf and root tissues, and the expression of ‘3875’ and ‘4800’ were also among the highest in leaves (Fig. 6a-b). In Duncan, ‘3875’ and ‘4800’ ranked as the highest expressed effectors, although the difference was not significant for ‘4800’ in roots compared with other effectors detected (Fig. 6c-d). The expression of Ca. *L. asiaticus* effector candidates were generally higher in the leaves of Cleopatra than in citron and Duncan, with top expressed candidates including ‘4800’, ‘3875’ and ‘5640’ in leaf and they were the only effectors with mRNA detected in the roots of Cleopatra (Fig. 6e-f).

## Discussion

The secretome of a pathogenic bacterium represents an array of molecules that play offensive roles during colonization, among which effectors are an important class of proteins capable of suppressing defense and/or manipulating host physiology. Whole genome sequencing of Ca. *L. asiaticus* indicated that this intracellular fastidious bacterium lacks Type III and IV secretion systems typical for extracellular pathogens, but maintains genes encoding the general secretory pathway/Sec-translocon [3], which was proposed to be the major pathway for Ca. *L. asiaticus* effector secretion [10]. Due to common features such as presence of secretion SP, effectors can be predicted computationally [38] and this accelerates the discovery of key virulence factors and targeted host resistance/susceptibility genes [39]. In this study, a total of 28 effector candidates were identified, by filtering Ca. *L. asiaticus* genome for presence of SP, absence of transmembrane domain and relatively small protein size (Additional file 1: Table S1). This list of candidates contains the effectors identified during our previous screening with different parameters [11] and was also discovered in an analysis for Sec-translocon-dependent proteins [10], confirming the commonality of bacterial effector features and reliability of the bioinformatics methods utilized in this study.

One important aspect of pathogen effectors study is to analyze the expression levels during the initial microbe-plant interactions. Effector candidates with high levels of transcript suggests an active protein utilization by the pathogen during the infection processes, which may provide guidance on the selection of effectors for further functional studies. To determine Ca. *L. asiaticus* expression by RT-qPCR, we used SSPs for cDNA synthesis and different sets of SSPs for qPCR (Additional file 2: Figure S1), which resulted in higher specificity in target amplifications and better performance for low abundance targets than using random primers for cDNA, or the same reverse SSPs for cDNA synthesis and qPCR (data not shown). As a result, a total of 20 primer sets (Additional file 3: Table S2) provided good Ca. *L. asiaticus*-specific amplification in multiple citrus genotypes including the relatively distant species *Poncirus trifoliata*. The expression of Ca. *L. asiaticus* effectors have been reported in several studies. For example, Ca. *L. asiaticus* effector expression profile was compared between infected citrus and ACP, and revealed interesting candidates differentially expressed in the two hosts [40]. In another study, semi-quantitative RT-qPCR detected the expression of *CLIBASIA_05315*, *CLIBASIA_00460*, *CLIBASIA_03230* and *CLIBASIA_05640* in several citrus types, with *CLIBASIA_05315* being promising as marker gene for disease early detection as it expressed in asymptomatic tissues [41]. However, because inoculation of bacteria was performed through grafting, plants materials in these studies were from relatively late infection stages which likely were at least several months following initial disease exposure [40]. Molecular events such as defense suppression by effectors and ETI-associated hypersensitive responses occur at early stages of contact, from hours to days after initial pathogen exposure [42]. Therefore we hypothesized that highly expressed Ca. *L. asiaticus* effectors during early host interactions are especially important for bacterial virulence, and their identification may lead to discovery of citrus resistance/susceptibility genes.

To test this hypothesis, we employed detached leaf inoculation to study effector expression within 7 days of initial bacterial contact (Fig. 2). Effective Ca. *L. asiaticus* inoculation was observed as soon as 6 h after infestation, resulting in a wide range of bacterial titers in multiple citrus genotypes (Fig. 2b-d). RT-qPCR analysis showed mRNA detection of only several effectors at various time points, which made it difficult to carry out mean comparisons for most of the genes. However, the expression of ‘4025’ in citron, and ‘5315’ in Duncan and Cleopatra were consistent at consecutive time points (Additional file 4: Table S3), suggesting possible roles during early host interactions, especially ‘5315’ which manipulates host cells for starch accumulation [12] and suppresses plant defenses [13]. Across all samples effector mRNA detection was titer-dependent, with higher number of effectors amplified in samples with higher bacterial quantification. This was further confirmed by analysis with greater sample sizes in citron, Duncan and Cleopatra (Fig. 3). This suggests that effector mRNA increases with bacterial cell number and thus effectors below the qPCR detection limit may become evident in citrus samples with high bacterial titer. It is also worth noting that the time points used in this study may not exactly correspond to ‘hours/days post inoculation (hpi/dpi)’ but rather to ‘inoculation access period (IAP)’ [36] within which inoculation occurs. This ongoing inoculation for seven days during our studies may explain ambiguous expression patterns of effectors in time point comparisons. Hence for simplicity we selected only 7-day IAP as representative of early bacterium-host interactions for the following studies.

To discover early and highly expressed effectors in citrus hosts with various HLB tolerance levels, six genotypes including citron, Duncan, Cleopatra, Pomeroy trifoliate, Washington navel, and Carrizo were subject to the detached leaf inoculation (7-day) by a common ACP population. Pair-wise comparisons ranked the effector expression levels to identify higher expressed candidates in each genotype (Fig. 4). Several effectors showed relative high expression in multiple genotypes, including ‘3695’ in citron, Cleopatra and Washington navel, ‘0460’ in citron, Washington navel, Pomeroy and Carrizo, ‘0420’ in citron and Pomeroy, and ‘4580’ in Cleopatra and Carrizo (Fig. 4a-f), suggesting these virulence factors are broadly active at the early infection stage. Among these effectors, the mRNA of ‘3695’ and ‘0460’ were detected at relatively high levels in both HLB-tolerant and susceptible citrus, suggesting they provide core virulence functions for bacterial colonization. Nevertheless, some effectors demonstrated tolerance/susceptibility-associated or host-specific high expression, including ‘0420’ in citron and Pomeroy, ‘4580’ in Cleopatra and Carrizo, ‘5320’ in Cleopatra, ‘4425’ in Duncan, ‘0525’ and ‘5315’ in Pomeroy, indicating host genetics may influence pathogen virulence factor expression. If confirmed, these tolerance- or resistance-associated effectors may be used as biomarkers to screen for HLB tolerance/resistance from citrus breeding materials. Further, the highly expressed effectors are good candidates for biochemical analysis to identify host binding targets, which may reveal important virulence mechanisms and lead to creation of resistant citrus.

A study was conducted using own-rooted citron, Duncan and Cleopatra plants that had been infected for more than a year, to evaluate effector expression in the pathogen after being fully established in tolerant and susceptible citrus hosts, assessing both leaf and root tissues (Fig. 5). Across all three citrus genotypes the expression patterns of effectors were similar between leaves but not roots (Fig. 6a-f). The number of effectors detected by RT-qPCR was less in roots than in leaves (Fig. 5d-e) which may be due to lower bacterial titers. Leaf expression of candidates ‘3875’, ‘4800’ and ‘5640’ were consistently among the highest regardless of citrus genotype/HLB tolerance levels (Fig. 6a, c and e). The expression profiles were markedly different from those of during the early infection for citron, Duncan and Cleopatra (Fig. 4a-c) which may suggest that Ca. *L. asiaticus* deploys different effectors over the time-course of infection. This is consistent with reports that this is a common strategy utilized by pathogens for successful colonization [15]. It is possible that our detached tissue-based study on early host interaction may be influenced by plant physiological changes associated with being detached from the mother plant. However, numerous researchers have employed the detached leaf inoculations to study outcomes of plant-pathogen interactions including accessing microbe pathogenicity and screening for resistant host and proved to produce consistent results to tests from whole plants [43–47]. In addition, at each sample time we also assessed mRNA for the citrus endogenous gene *UPL7* and the expression was similar at all of time points and across all genotypes, suggesting metabolism was not compromised throughout the study period. In roots, high mRNA abundance of ‘5640’ in citron and ‘3875’ in Duncan and Cleopatra, even at low bacterial titers, may be evidence that their functions may contribute to HLB-related root damage such as root starch depletion and dieback reported to be causal rather than the consequence of HLB disease [37, 48]. Taken together, this part of the study identified effector candidates showing higher expression following established colonization in both HLB tolerant and susceptible citrus. The functional analysis of these candidates may enhance understanding of bacterial virulence and host interactions during chronic Ca. *L. asiaticus* infection.

## Conclusions

A group of 28 candidate effectors were identified from the Ca. *L. asiaticus* genome via bioinformatics. Their transcriptional levels were studied in the infected citrus leaves exposed to ACP within 7 days from susceptible Duncan grapefruit and Washington navel orange, tolerant citron and Cleopatra mandarin, and resistant Pomeroy trifoliate and Carrizo citrange. Candidate effectors *CLIBASIA_03695*, *CLIBASIA_00460*, *CLIBASIA_00420*, *CLIBASIA_04580*, *CLIBASIA_05320*, *CLIBASIA_04425*, *CLIBASIA_00525* and *CLIBASIA_05315* showed relatively high expression in host-specific or -nonspecific manners. In citrus with HLB exposure for over one year, the expression of effectors was compared between leaves and roots tissues and indicated relatively high expression of *CLIBASIA_03875*, *CLIBASIA_04800* and *CLIBASIA_05640* in all leaf and some root tissues of citron, Duncan and Cleopatra genotypes. The identified Ca. *L. asiaticus* effectors candidates with high temporal and spatial expression levels may have roles in early bacterial colonization, host tolerance suppression and long-term survival that will be subject to further functional studies.

## Methods

### Plant materials

All of the plant materials were clonally produced and maintained at the US Horticultural Research Lab greenhouse facilities. Healthy seedlings of Duncan grapefruit, Cleopatra mandarin, citron, Pomeroy trifoliate, Washington naval orange and Carrizo citrange were grown in pots in the greenhouse that were watered daily and fertilized weekly. Horticultural mineral oil was applied as needed to control pests but no other insecticides were applied. For detached leaf inoculation, leaves for each citrus type were collected at similar maturity and size, rinsed and used immediately for described experiments. The leaf and root analysis used citron, Duncan and Cleopatra plants derived from seed and inoculated more than a year earlier, verified as Ca. *L. asiaticus*-infected, and maintained in the quarantine greenhouse.

### Detached leaf inoculation and sample collection

The leaf samples were collected between 10 am to noon, gently rinsed and dried in the lab, with the petioles placed in water in 1.5 mL centrifuge tubes sealed with Parafilm. Each detached leaf, with its petiole in water, was placed in a 50 mL plastic centrifuge tube with a mesh top and was exposed to 10 psyllids collected from colonies maintained on Ca. *L. asiaticus* infected plants. Tubes containing leaves and psyllids were kept on the lab bench with supplemental light of 16 h per day. The light set up is previously described with light intensity at 9687 ± 3460 lm/m^2^ [49]. At the time of destructive sampling, the ACP were removed by vacuum and the leaves were wiped clean before sampling. A group of 10–15 leaves were collected each at 6 h, 1d, 3d and 7d post ACP infestation, or for each citrus genotype at the 7d time point. The midrib tissues were excised for PCR quantification of Ca. *L. asiaticus 16 s* rDNA, and the remaining leaf tissues were placed initially in liquid nitrogen and then stored at − 80 °C for RNA isolation and expression analysis.

### Whole plant inoculation and sample collection

Ca. *L. asiaticus*-infected citron, Duncan and Cleopatra plants, all own-rooted from seed, were inoculated by ACP infestation during a previous study in 2016 and maintained in a greenhouse along with uninfected controls [9]. Briefly, six citrus plants with young flushes were infested by 400 Ca. *L. asiaticus* positive ACP or negative ACP (as mock inoculation) in dome cages for 2 weeks. Then insects were removed by aspiration, and plants were treated with carbaryl insecticide and greenhouse growth condition were resumed. A pool of 4 to 6 leaves were collected from each plant and used for leaf analysis of effector expression. Fibrous roots were sampled peripherally from the root mass of each plant, rinsed and stored at − 80 °C for analysis.

### Determination of Ca. *L. asiaticus* bacterial titer

To quantify Ca. *L. asiaticus* in citrus leaves, DNA was isolated from midrib of leaves or fibrous roots using DNeasy plant mini kit (Qiagen, Gaithersburg, MD, USA), and the quantity and quality were determined by a NanoDrop Spectrophotometer (Thermo Scientific, Wilmington, DE, USA). For each sample, 100 ng of DNA was used for qPCR of Ca. *L. asiaticus 16 s* rDNA using Las Long primers and SYBR chemistry (Thermo Fisher Scientific, Waltham, MA, USA) and an ABI7500 thermal cycler (Applied Biosystems, Foster City, CA, USA). The threshold cycle (C_t_) values were used to calculate bacterial titer using the standard curve method [9].

### Expression analysis of effector candidates by RT-qPCR

The total RNA was isolated from Ca. *L. asiaticus* positive leaf or root samples using TriZol reagent (Invitrogen, Carlsbad, CA, USA) following the manufacturer’s instructions. On-column DNase treatment and RNA purification were performed using RNeasy Plant Mini Kit (Qiagen). The quantity and quality of RNA were determined using a NanoDrop Spectrophotometer (Thermo Scientific). Subsequently, 1 μg of RNA was used for cDNA synthesis by QuantiTect Reverse Transcription Kit (Qiagen). A mix of SSPs (Additional file 3: Table S2) targeting Ca. *L. asiaticus* effectors, *16 s* and a citrus endogenous gene *UPL7* was used for cDNA synthesis at a final concentration of 0.5 μM each. A 20 μL of reverse transcription reaction for each sample was performed according to the kit protocol. Subsequently, the cDNA was diluted to 5 ng/μL and 2 μL was used in each qPCR reaction together with forward and reverse SSPs (Additional file 3: Table S2) to amplify effector candidates.

## Additional files

Additional file 1: **Table S1.** The list of putative effectors identified by filtering Ca. *L. asiaticus* genome using bioinformatics. Supplemental Table 1-A contains the list of Ca. *L. asiaticus* genes with a signal peptide predicted by SignalP. Supplemental Table 1-B contains the list of Ca. *L. asiaticus* genes having signal peptide but not transmembrane domain, with the protein size smaller than 200 amino acids. (XLSX 22 kb)

Additional file 2: **Figure S1.** The design of primers for reverse transcription (RT) and qPCR amplifying Ca. *L. asiaticus* effector candidates. The illustration uses *CLIBASIA_05315* sequence as an example, where the RT primer (yellow) was selected towards the 3′ end of the sense strand (reverse complement to the selected), and the forward (red) and reverse (blue, reverse complement to the selected) primers for qPCR were located downstream along the cDNA synthesis direction. (DOCX 19 kb)

Additional file 3: **Table S2.** Primer sequences for reverse transcription and qPCR analysis of effector expression. The list of primers used in this study for reverse transcription and qPCR (XLSX 10 kb)

Additional file 4: **Table S3.** A time course study on the expression of Ca. *L. asiaticus* effectors in citron, Duncan and Cleopatra using the detached leaf assay. Three randomly selected Ca. *L. asiaticus*-infected samples were collected for each time point (Fig. 2) and were analyzed for expression by RT-qPCR. The data was presented as relative expression levels, based on normalization with citrus *UPL7* (delta Ct), and transformed into 2^-ΔCt^. The table was formatted with color scales using Excel where values increase from green to red colors. The two highlighted columns were the two samples with high bacterial titer and larger number of detected candidate effectors. (XLSX 15 kb)

## Abbreviations

ACP: Asian citrus psyllid
*Ca.* L. asiaticus: *Candidatus* Liberibacter asiaticus
C_t_: Cycle threshold
ETI: Effector-triggered immunity
ETS: Effector-triggered susceptibility
HLB: huanglongbing
hpi/dpi: Hours/days post inoculation
IAP: Inoculation access period
NCBI: National Center for Biotechnology Information
PAMPs: Pathogen-associated molecular patterns
PCR: Polymerase chain reaction
RT-qPCR: Reverse transcription quantitative PCR
SP: Signal peptide
SSPs: Sequence specific primers
