# Supplementary material for: Temporal and spatial detection of Candidatus Liberibacter asiaticus putative effector transcripts during interaction with Huanglongbing-susceptible, −tolerant, and -resistant citrus hosts
Source: BMC Plant Biol. 2019 Apr 2;19:122. doi: 10.1186/s12870-019-1703-4 (PMC6444692; doi:10.1186/s12870-019-1703-4)
Supplement: Supplementary file 2 — Figure S1. The design of primers for reverse transcription (RT) and qPCR amplifying Ca. L. asiaticus effector candidates. The illustration uses CLIBASIA_05315 sequence as an example, where the RT primer (yellow) was selected towards the 3′ end of the sense strand (reverse complement to the selected), and the forward (red) and reverse (blue, reverse complement to the selected) primers for qPCR were located downstream along the cDNA synthesis direction. (DOCX 19 kb) [file 12870_2019_1703_MOESM2_ESM.docx]

>CLIBASIA_05315

GTGCGTAAAAATTTATTAACCTCAACCTCATCTTTAATGTTTTTTTTCTTATCTTCTGGCTATGCTTTATCTGGCAGTA

GTTTTGGTTGTTGTGGAGAATTTAAAAAGAAAGCTTCTTCACCTAGAATCCATATGCGTCCTTTCACCAAGTCATCA

Forward

CCTTATAACAACTCAGTGAGTAATACAGTGAATAATACTCCGCGTGTTCCTGATGTCTCTGAAATGAACAGCTCT

AGGGGTTCTGCTCCTCAATCTCATGTTAATGTTTCTTCTCCTCATTATAAACATGAATACAGTTCTTCTTCGGCATCTT

RT direction

Reverse

CTTCAACACATGCTTCGCCTCCTCCTCATTTTGAACAGAAGCACATTAGTCGCACTCGTATTGACTCAAGCCCTC

ACCCGGTCATATTGATCCTCATCCCGATCATATTAGAAATACACTTGCACTCCATAGAAAAATGTTGGAGCAGTCTT

GA

Supplemental figure 1. The design of primers for reverse transcription (RT) and qPCR amplifying *Ca*. L. asiaticus effector candidates. The putative effector sequences were obtained from the *Ca*. L. asiaticus psy62 genomic database. The figure uses CLIBASIA_05315 as an example, where the RT primer (yellow) was selected towards the 3' end of the sense strand (reverse complement to the selected), and the forward (red) and reverse (blue, reverse complement to the selected) primers for qPCR were located downstream along the cDNA synthesis direction.
